# Biodiversity pressure from fruit and vegetable consumption in the United Kingdom, India and South Africa varies by product and growing location

**DOI:** 10.1038/s43016-025-01222-y

**Published:** 2025-08-25

**Authors:** Abbie S. A. Chapman, Rosemary Green, Genevieve Hadida, Harry Kennard, Tafadzwanashe Mabhaudhi, Pauline Scheelbeek, Carole Dalin

**Affiliations:** 1 Institute for Sustainable Resources, University College London, London, UK; 2 Centre for Biodiversity and Environment Research, Department of Genetics, Evolution and Environment, University College London, London, UK; 3 Faculty of Epidemiology and Population Health, London School of Hygiene and Tropical Medicine, London, UK; 4 Centre on Climate Change and Planetary Health, London School of Hygiene and Tropical Medicine, London, UK; 5 University of Texas at Austin, Austin, TX USA; 6 Centre for Transformative Agricultural and Food Systems, School of Agricultural, Earth & Environmental Sciences, University of KwaZulu-Natal, Pietermaritzburg, South Africa; 7 Centre for Water Resources Research, School of Agricultural, Earth and Environmental Sciences, University of KwaZulu-Natal, Scottsville, South Africa; 8 Laboratoire de Géologie, École normale supérieure, CNRS, PSL Université, IPSL, Paris, France

**Keywords:** Agroecology, Biodiversity, Environmental impact, Sustainability, Agriculture

## Abstract

In many countries around the world, fruit and vegetable consumption must increase to improve human health, potentially pressuring local and global biodiversity. Here we use biodiversity-pressure metrics to compare the biodiversity pressures associated with fruits and vegetables consumed in the United Kingdom, India and South Africa. We found that biodiversity pressure for individual crops varies greatly with origin. In all three countries, imported fruits are typically associated with greater pressure than domestically grown fruits. Contrastingly, in India and South Africa, imported vegetables generally have a lower biodiversity pressure than domestically grown vegetables. Oranges, popular in the United Kingdom and South Africa, exert almost three times more biodiversity pressure than bananas—one of the most-consumed fruits in the United Kingdom and India. Our analysis illustrates the quantification of crop-specific biodiversity pressures and provides evidence for the development of more sustainable food systems.

## Abstract (web summary)

Increasing the consumption of specific food groups will be key for human health but might also lead to environmental impact. This study compares the biodiversity pressures associated with fruits and vegetables consumed in the United Kingdom, India and South Africa.

## Main

Current agrifood systems are both fundamental and damaging to planetary health^1^. Researchers have sought to identify presently unused land suitable for agriculture to meet the nutritional needs of the growing global human population and increasing consumption in some countries^2–4^. Nonetheless, agricultural land use is the leading driver of biodiversity loss^5–8^. Declines in biodiversity may reduce crop yields over time because biodiversity supports food production via ecosystem services, such as pest control and animal-mediated pollination^9–13^. For planetary health, and to meet international sustainability and biodiversity agreements^14,15^, we must find ways to ensure a stable supply of healthy foods for human populations while mitigating environmental damage^16,17^.

Shifting to a more plant-based diet is an established demand-side climate change mitigation strategy and can reduce other food-system pressures on the environment, such as water stress^18^, carbon^19,20^ and land-use change^21,22^. The impacts on biodiversity are less well quantified. Although the appropriateness of some proposed dietary shifts is context dependent, there is no doubt that increasing fruit and vegetable consumption has positive human-health outcomes^23^. Eating insufficient fruits and vegetables has been linked to a higher risk of cardiovascular disease and some cancers^23^. Governments worldwide have therefore set guidelines to encourage people to eat enough fruits and vegetables daily^24^. Nevertheless, many countries are not meeting their targets, with most people failing to consume the 400 g day^−1^ advised by the World Health Organization^25^.

Because promoting increased fruit and vegetable consumption is an important ‘win’ for human health, it is important to consider the impact that increased consumption (and thus production) of these crops might have on biodiversity. Previous research has emphasized trade-offs between biodiversity and cropland. Much of this research has focused on deforestation, land area and general agricultural land uses^3,26–28^, rather than specific crops^7,29^. Research focusing on specific crops^30–32^ has associated arable lands used for ‘staple’ (for example, maize, rice) and export-intensive ‘cash’ crops (for example, oil palm, soybeans) with fewer species than natural lands^7^. Global-scale environment–food studies have typically focused on environmental impacts aside from biodiversity^17,22,33^, or quantified biodiversity impacts with an overall loss value per region^34^ and/or land-use type^3^, rather than measuring spatial variation (although see Lenzen et al.^32^ for export-intensive crops linking species threat and trade, Chaudhary and Kastner^35^ for a complementary study estimating species committed to extinction due to crop and trade partnerships, Boakes et al.^36^ for a regional assessment of the land- and greenhouse-gas-driven footprints of food on biodiversity, and Schneider et al.^37^ for an analysis considering changes to biodiversity intactness and impacts on biodiversity hotspots associated with cropland expansion scenarios). As well as a relative lack of horticultural studies^35,38^, assessments of the sustainability of different diets tend to focus on greenhouse gases, and, more recently, also on water stress (for example, due to irrigation of fruit trees in South Africa^39^), or fertilizer and pesticide use^17,22,33,40,41^. Research measuring greenhouse gases, land and water use, and eutrophication potential associated with different foods has shown that switching to healthier, more plant-based diets may have positive outcomes for the environment, but this mostly focuses on protein sources rather than fruit and vegetable nutrient delivery^40,42^. Fruits and vegetables have, however, been associated with low greenhouse gas emissions relative to other foods^43–45^, and vegetables have lower greenhouse gas and water-use impacts than fruits^46^.

Overall, there remains a relative lack of understanding of the potential biodiversity impacts of producing specific, commonly consumed fruits and vegetables around the globe (for example, fruits, vegetables and nuts are considered together^47^). We also do not know where potential biodiversity pressures could be greatest when accounting for the role of international trade and consumption because previous research typically focuses on the supply-side of the food system (although see refs. ^32,35^). It remains to be seen how biodiversity pressures associated with fruits and vegetables vary across countries, how much this is influenced by consumption (demand-side) and where the ‘hotspots’ of biodiversity pressure are for these foods. To investigate this, spatialized metrics of biodiversity pressure are needed.

In this study, we quantify the link between healthy ecosystems and healthy people (planetary health) by developing spatially explicit and crop-specific biodiversity-pressure metrics, which can be measured globally. Our metrics could be implemented into integrated assessment models. These metrics provide an alternative, complementary method to the approaches in Lenzen et al.^32^, Molotoks et al.^48^, Boakes et al.^36^ and Chaudhary and Kastner^35^, enabling us to quantify the potential impact of each unit of crop consumed in a country on species in that country and its trade partners, facilitating comparisons across locations and crops. Unlike many land-based indicators of biodiversity, we use an explicit biodiversity metric. We expect biodiversity pressures of fruits and vegetables to vary according to: (1) the composition of the mix of fruits and vegetables consumed in a country, affecting local biodiversity and crop yields in growing areas; (2) the origin of supply to a country, determining the domestic and international biodiversity pressures associated with national diets; and (3) the volumes produced and consumed, influencing farming practices needed and their impacts.

The metrics presented here enable us to quantify the influence of these different factors ((1)–(3)) on the biodiversity pressures associated with fruit and vegetable consumption (specifically the production this consumption drives). For our work, we must assume that biodiversity and crop coexistence creates pressure that would not be present if land was uncultivated. This assumption is well supported based on previous studies comparing biodiversity associated with different land-use categories and land-use changes^7,48–50^. We focus our study on the United Kingdom, India and South Africa—three countries with strong trade relationships in fruit and vegetables but differing levels of self-sufficiency. We quantify and compare the biodiversity pressures associated with the consumption of 50 different fruits and vegetables, supplied via domestic production and imports, although our approach can be used to study other crops. We identify crops and countries with higher biodiversity pressure and consider the influence of trade on the sustainability of the consumption of healthy foods in the United Kingdom, India and South Africa.

## Results

### Biodiversity pressures associated with fruit and vegetable production are global in scale

**Fig. 1:**
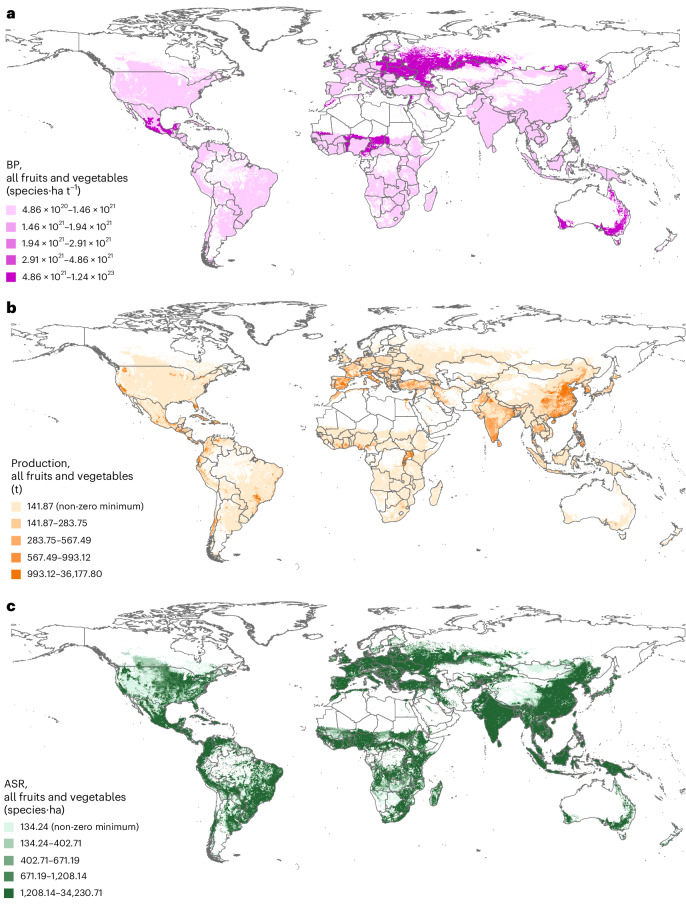
Global maps of all fruits and vegetables analysed. **a**–**c**, Maps showing BP (range (maximum value minus minimum value) across all countries: 8.34 × 1,023 species·ha t^−1^) (**a**); production (after Monfreda et al.^79^ and Ramankutty et al.^80^) (range across all countries: 16,060 t); and ASR (range across all countries: 1,321,752 species·ha) (**c**). Country administrative boundary data were sourced from the World Bank^92^, but these maps show values per grid cell. Some disputed areas and countries without UN representation may not be shown on these maps. Maps are projected in the Geographic Coordinate System WGS 1984 and cells are 5-arcmin resolution (∼1,000 ha at the equator). Global rankings of fruits and vegetables according to BP, production and ASR are provided in Supplementary Table 1.1. Mean values of BP per crop per country are provided in Supplementary Table 1.2. Base map data from The World Bank under a Creative Commons license CC BY 4.0.

The biodiversity pressure (BP, species·ha t^−1^, Fig. 1a) associated with a cultivated crop is the ratio between the affected species range (ASR, species·ha, Fig. 1c) and the crop production (t, Fig. 1b). The ASR (equation (1), Methods) is the number of species distributions overlapping with a cultivated area. Overall, the biodiversity pressure captures the number of vertebrate species potentially impacted by a given crop over a certain area, per unit of crop produced (equations (2) and (3), Methods). The biodiversity pressure associated with national production is a sum of the biodiversity pressure over a country, multiplying the pressure by a country’s production (equation (4), Methods). Fruit and vegetable production in Nigeria, Burundi, Rwanda, India and Brazil affects the greatest proportion of species ranges (Fig. 1c and Supplementary Table 1.1). For example, the mean ASRs for Nigeria, summed across all fruits and vegetables produced there, totals 1,823,654 species·ha (Supplementary Table 1.1). By the nature of the index and underlying data, this does not mean that nearly two million species are affected. The number can, however, be interpreted relative to other countries. For example, the country with the lowest above-zero value for the mean ASR summed over all fruits and vegetables produced is Ireland, with 1,083 species·ha (Supplementary Table 1.1). The United Arab Emirates, Bahrain and Tunisia are the countries with the greatest total production (Supplementary Table 1.1), exceeding 4,000 dry-weight tonnes of fruits and vegetables, with this also affecting a relatively high proportion of species ranges (ranking 13th, 50th and 7th of 169 producing countries for mean ASR per country, respectively; Supplementary Table 1.1). Of these countries, however, only Tunisia is one of the highest-ranking countries for mean per-tonne biodiversity pressure associated with its fruit and vegetable production, ranking 6th of 169 producing countries (Supplementary Table 1.1). Countries in North Africa and the Middle East, and Australia and parts of eastern Europe, produce the commodities with highest biodiversity pressure per tonne grown (Fig. 1a). Fruits and vegetables produced in the Congo and Australia have the greatest total per-tonne biodiversity pressure of all countries analysed, when values per grid cell (shown in Fig. 1) are summed across each country for all fruits and vegetables (Supplementary Table 1.1).

### Trade influences biodiversity pressure

**Fig. 2:**
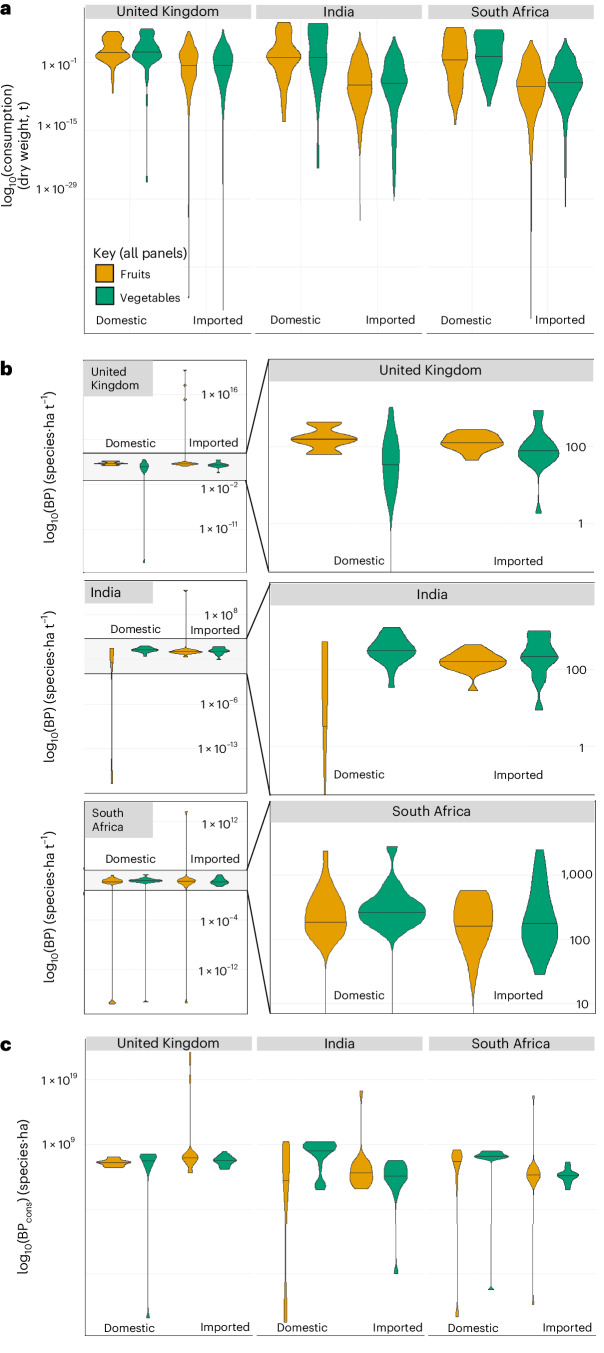
Pressures associated with fruits and vegetables consumed by the United Kingdom, India and South Africa (supplied via domestic production (‘domestic’) or imports (‘imported’)). **a**, Dry-weight consumption (t, tonnes). **b**, Per-tonne BP (species·ha t^−^^1^, species·hectares per tonne), with the weighted-average BP for each imported product included for the imported components. **c**, BP associated with national consumption (BP_cons_; species·ha, species·hectares), measuring the overall impact of all tonnes consumed by a country. Panel **b** shows paired facets per country, with the right-hand images being ‘zoomed in’ versions of the left, for visualization purposes. This figure summarizes the values for each fruit and vegetable analysed, for all values greater than zero. The violin plots therefore show the distribution for 21, 49 and 40 domestically produced fruits and vegetables, and 50, 48 and 45 imported fruits and vegetables, for the United Kingdom, India and South Africa, respectively. A log_10_ transformation has been applied for visualization purposes. Untransformed median values are provided in Supplementary Table 1.4, together with mean values and proportions of fruits and vegetables supplied via domestic production and imported.

The biodiversity pressure associated with national consumption (equation (5), Methods) accounts for the pressures embedded in the consumption of a food by a country, whether the food is sourced domestically or via imports. Overall, the biodiversity pressure associated with the national consumption of countries will vary substantially according to the trade partners supplying their fruits and vegetables. For instance, India and South Africa have a larger proportion of their biodiversity pressures originating from domestic production compared with the United Kingdom, and the United Kingdom ‘outsources’ orders of magnitude more biodiversity pressure than the other focal countries (Fig. 2a and Supplementary Table 1.4). For example, the mean pressure associated with national consumption of fruits produced in the UK is 2.7 × 10^6^ species·ha, whereas the same measure for imported fruits is 6.5 × 10^21^ species·ha (Fig. 2a). However, all three countries have high biodiversity pressures associated with their imports (Fig. 2a and Supplementary Table 1.4).

In India and South Africa, median biodiversity pressure tends to be higher for imported fruits than for fruits supplied domestically, although the opposite is true for vegetables (Fig. 2b,c and Supplementary Table 1.4). The United Kingdom is the least self-sufficient of the three focal countries, getting the greatest share of its fruit and vegetable supply via imports (Fig. 2a and Supplementary Table 1.4). The United Kingdom’s biggest fruit and vegetable imports are tomatoes from Italy, Spain, Greece and Portugal, grapes from Turkey, and apples from France. Overall, biodiversity pressure varies by crop and location more than by food group (that is, fruits versus vegetables) (Fig. 2).

### Biodiversity pressure is crop and location specific

**Fig. 3:**
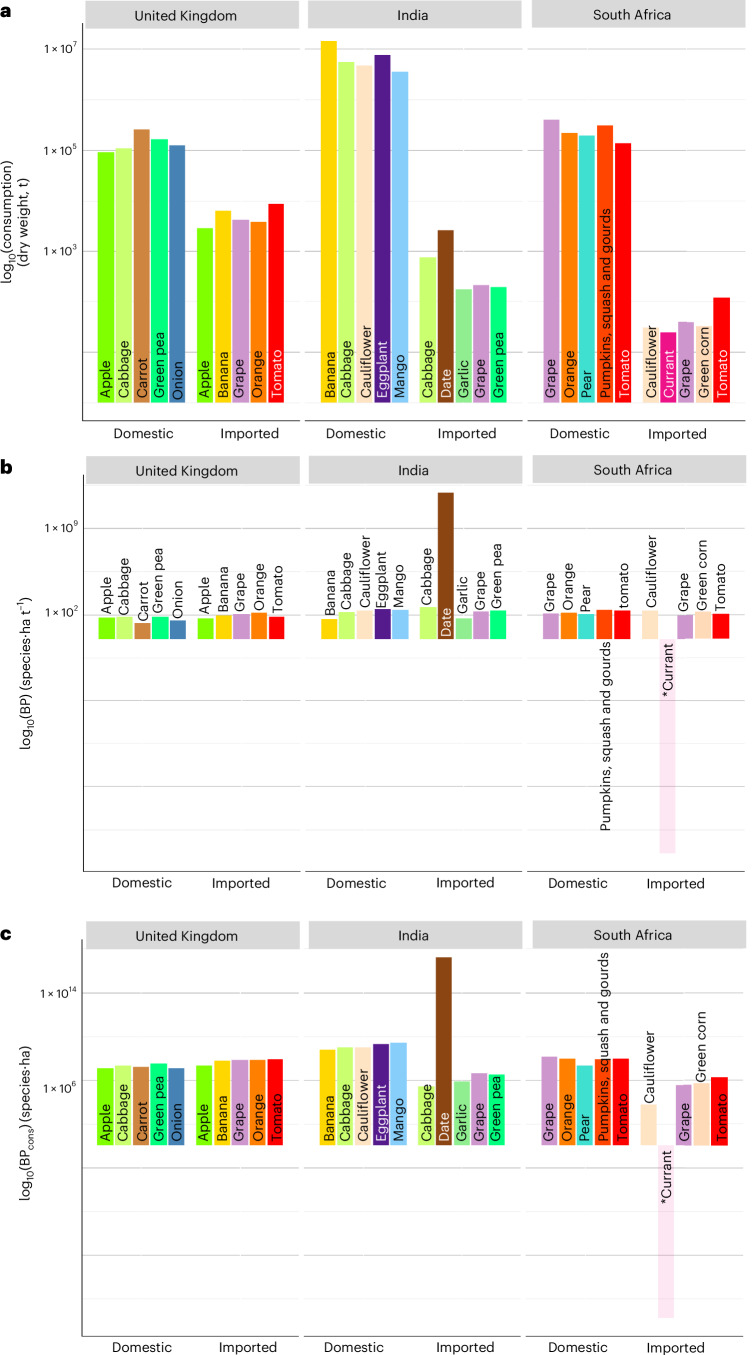
Pressures associated with specific fruits and vegetables consumed by the United Kingdom, India and South Africa. **a**–**c**, The top five crops according to consumption per country in each crop group (supplied via domestic production (‘domestic’) or imports (‘imported’)): dry-weight consumption (tonnes) (**a**), per-tonne BP (species·hectares per tonne), with the weighted-average BP for each imported product included for the imported components (**b**), and the BP associated with national consumption (BP_cons_, species·hectares), measuring the overall impact of all tonnes consumed by a country (**c**). Values for all fruits and vegetables analysed are provided in Supplementary Tables 1.1–1.3. A log_10_ transformation has been applied for visualization purposes; the tinted bar and asterisk (*) next to ‘currant’ highlight that this fruit has a positive value, but this is very small and therefore presents as a negative number after the log_10_ transformation. This figure is complementary to Fig. 2, which shows violin plots of the distribution of pressures and consumption of all fruits and vegetables consumed in each focal country; here, we show the pressures and consumption values for some of the crops summarized in Fig. 2.

Bananas are the most-consumed domestically produced fruit in India and yet they do not have the highest biodiversity pressure associated with national consumption (Fig. 3a,c) or per-tonne pressure (Fig. 3a,b) of the most-consumed fruits and vegetables in this country. This is also the case for carrots consumed and produced in the United Kingdom, but not for grapes consumed and produced in South Africa, which do have the greatest biodiversity pressure associated with national consumption of fruits and vegetables consumed in South Africa (Fig. 3a,c). The most-consumed imports in the United Kingdom and South Africa are tomatoes and, in India, dates (Fig. 3a). South Africa’s imported tomatoes and India’s imported dates have the greatest consumption-based biodiversity pressure of the fruits and vegetables imported by each country, with dates the highest of all (Fig. 3c). Although imported tomatoes have a high pressure associated with national consumption in the United Kingdom, the pressure is similar to that of the United Kingdom’s imported oranges, grapes and bananas (Fig. 3c).

Several fruits and vegetables stand out as particularly high pressure across all three countries. For instance, despite differing consumption (Fig. 3a), grapes, green peas, oranges and tomatoes have a high per-tonne biodiversity pressure, and a high biodiversity pressure associated with national consumption, in multiple focal countries (Fig. 3b,c). Nevertheless, overall, biodiversity pressure is crop and location specific.

### Origin affects pressure more than crop

**Fig. 4:**
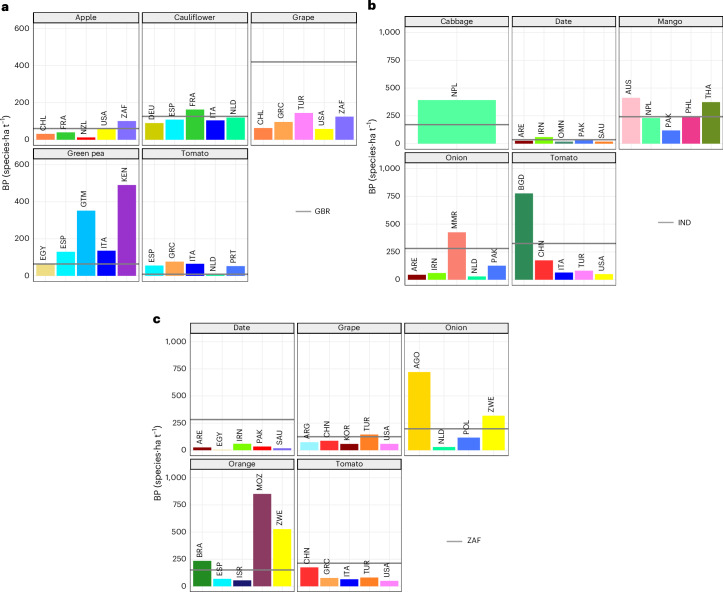
The biodiversity pressure of the crops with the highest biodiversity pressure associated with national consumption (BP_cons_), which are produced domestically and imported. **a**–**c**, Biodiversity pressure data for the United Kingdom (**a**), India (**b**) and South Africa (**c**). The grey line represents the biodiversity pressue of the focal country, for comparison with the top five trade partners supplying the given crop, shown with coloured bars. Countries with bars above the grey, domestic line represent production under a stronger biodiversity pressure than that of the domestic focal country, and vice versa. The *y*-axis values are labelled in the left-most panels for all plots in the panel. Where crops were not imported and could not be compared to domestically produced crops, the crops with the next greatest BP_cons_ are shown (India: eggplants, pumpkins, cauliflowers, okra; South Africa: pumpkins; United Kingdom: sour cherry, plantain, dates, oranges, bananas). Country names have been abbreviated using ISO (International Organization for Standardization) alpha-3 codes as follows (in order of appearance): CHL, Chile; FRA, France; NZL, New Zealand; USA, United States; ZAF, South Africa; DEU, Germany; ESP, Spain; ITA, Italy; NLD, Netherlands; GRC, Greece; TUR, Turkey; EGY, Egypt; GTM, Guatemala; KEN, Kenya; PRT, Portugal; GBR, United Kingdom; NPL, Nepal; ARE, United Arab Emirates; IRN, Iran; OMN, Oman; PAK, Pakistan; SAU, Saudi Arabia; AUS, Australia; PHL, Philippines; THA, Thailand; MMR, Myanmar; BGD, Bangladesh; CHN, China; IND, India; ARG, Argentina; KOR, Republic of Korea; AGO, Angola; POL, Poland; ZWE, Zimbabwe; BRA, Brazil; ISR, Israel; MOZ, Mozambique. Mean BP_cons_ values per crop are provided for each focal country in Supplementary Table 1.3.

The biodiversity pressures of fruits and vegetables vary more by origin than by crop (Figs. 3 and 4). For instance, tomatoes are a relatively high-pressure food imported by all three focal countries (Fig. 3) with different import-associated pressures in each country (Fig. 4). South Africa has a greater biodiversity pressure associated with its domestically grown tomatoes than any of its five dominant international suppliers, whereas the opposite is true of the United Kingdom (Fig. 4a,c). Bangladesh is the only major trade partner of India with a greater per-tonne biodiversity pressure (776 species·ha t^−1^) for tomatoes than India’s production (326 species·ha t^−1^; Fig. 4b). These contrasting situations for the same crop—tomato—illustrate that domestic production does not always result in a lower biodiversity pressure than importing because the biodiversity pressure depends on the amounts being produced, and where, with respect to local vertebrate species richness. This point is further emphasized by examples such as cauliflowers and grapes grown and consumed in the United Kingdom instead of being imported from trade partners such as Germany, Chile and the United States, where the per-tonne biodiversity pressure of these crops is lower (Fig. 4a). Similarly, Indian onions and mangoes have a higher per-tonne biodiversity pressure than those imported from Pakistan, and dates and tomatoes show lower pressure when imported from most other major trade partners (Fig. 4b). Contrastingly, South African grapes have a lower per-tonne biodiversity pressure in South Africa than grapes imported from Turkey (Fig. 4c).

**Fig. 5:**
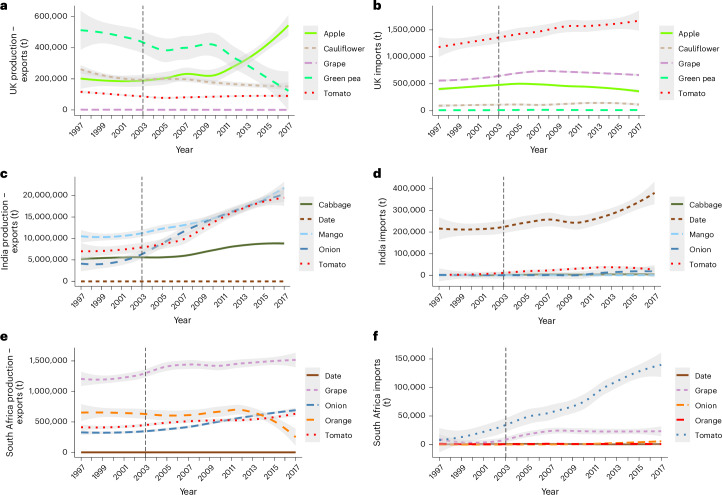
Import and production trends for the crops with the highest biodiversity pressure associated with national consumption (BP_cons_) which are produced domestically (production − exports) and imported for consumption. **a**–**f**, Import and production of domestically produced crops (**a**,**c**,**e**) and crops inported for consumption (**b**,**d**,**f**) by the United Kingdom (**a**,**b**), India (**c**,**d**) and South Africa (**e**,**f**). Note that these fruits and vegetables are comparable with those presented in Fig. 4, to identify whether fruits and vegetables which have a lower biodiversity pressure when grown domestically have been increasingly produced domestically since 2000, or whether imports have increased despite their higher associated pressure, and vice versa. The grey shading indicates 95% confidence intervals.

The data we analysed represent the year 2000. To understand changes since then, we analysed imported and domestically sourced supply (production minus export) trends for each focal country and the fruits and vegetables shown in Fig. 4 (Fig. 5). Since 2000, some fruits and vegetables with a generally lower per-tonne domestic biodiversity pressure are increasingly being produced domestically (for example, Indian cabbages and South African oranges; Fig. 5). However, some fruits and vegetables with a lower domestic biodiversity pressure have been increasingly supplied by imports since 2000 (for example, tomatoes in the United Kingdom; Fig. 5). In general, the United Kingdom imports more fruits and vegetables than it produces (Fig. 5).

### Spatialized approaches reveal within-country pressure variation

**Fig. 6:**
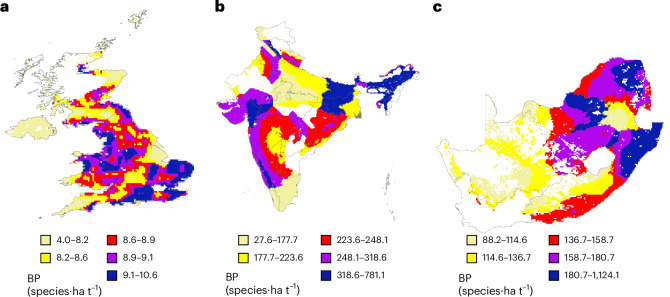
Biodiversity pressure of examples of the most-consumed fruit/vegetable crops supplied by domestic production and imports. **a**–**c**, The biodiversity pressure of some examples of the most-consumed fruit/vegetable crops supplied by domestic production and imports in each focal country, shown in Fig. 4 to be potentially placing lower pressure on biodiversity when produced domestically rather than imported: tomatoes in the United Kingdom (**a**), cabbages in India (**b**) and oranges in South Africa (**c**). Note that some of the high-pressure areas are in critical biodiversity hotspots (Extended Data Fig. 1) and the colour spectrum indicates different BP values in each panel.

The biodiversity pressure also varies spatially within a given country for the same crop (Fig. 6). For instance, each country has ‘hotspots’ of relatively high biodiversity pressure for each example crop in Fig. 6. In the United Kingdom and India, tomato and cabbage biodiversity pressure hotspots are spread across most regions of each country (Fig. 6a, b), whereas South African oranges have greatest biodiversity pressures in eastern regions (Fig. 6c).

## Discussion

In addition to proposing measures of biodiversity pressure that can assist with sustainability assessments, our analysis quantifies the influence of crop, origin and volumes produced and consumed on the biodiversity pressures associated with fruits and vegetables consumed in the United Kingdom, India and South Africa. All three factors have a strong influence on biodiversity pressure, but the origin and consumption volumes are key because even the same crops have different pressures in each focal country (for example, tomatoes have a pressure of 9 species·ha t^−1^ in the United Kingdom versus 326 species·ha t^−1^ in India, and grapes have a pressure of 125 species·ha t^−1^ in South Africa compared with 420 species·ha t^−1^ in the United Kingdom). We also identify some outliers, such as persimmons, which have several orders of magnitude higher per-tonne biodiversity pressure than other fruits and vegetables because they are produced with relatively low yields (low production per hectare) in high biodiversity areas. Our work highlights complexities not always evident in previous research, which grouped fruits and vegetables^47^, measured biodiversity impacts by land-cover class^7^, or summarized analysis over large ecological systems^3^. Here, consumption data enable us to begin to incorporate complexities such as national dietary preferences in assessments of the biodiversity pressures of different foods, accounting for the amounts of different fruits and vegetables consumed in each country and where they are sourced from.

The United Kingdom, India and South Africa all had a greater per-tonne biodiversity pressure associated with their imports than with their domestic production of fruits (and the same in the United Kingdom for vegetables), when pressure was measured as an average of all fruits and/or vegetables consumed between 1997 and 2003. However, biodiversity pressure associated with national consumption was greater for domestically produced vegetables than for those imported to the United Kingdom, India and South Africa. These findings could be interpreted to suggest that domestic production is a major source of pressure, putting the future sustainability of vegetable supplies at risk in these countries. Instead, combining information from various measures reveals that biodiversity pressure is specific to crop and country of origin. Here, we show that biodiversity pressures are not always greater for domestic fruits or vegetables than for imported supplies.

Nevertheless, because countries such as the United Kingdom—unable to grow some fruits and vegetables at commercial scale under present climatic conditions—‘outsource’ a substantial amount of the biodiversity pressure associated with their fruit and vegetable consumption, the national dietary policies of these import-dependent countries have international consequences for biodiversity. This is of particular importance as local food crop production alone cannot fulfil future food demands^51^. We therefore need conscientious decision-making from importing countries to ensure sustainable futures for both producing and importing countries. Many of the areas with lower biodiversity pressures in 1997–2003 (for example, western Europe, the United States and Canada) may simply have a lesser impact because the biodiversity baseline is already lower due to historic human impacts on biodiversity—a common limitation of biodiversity records^52^. As has been established in recent climate-change agreements^53^, it will be important for environmental and equity reasons, including social justice, to ensure that food security in high-income countries with ‘less to lose’ with respect to threatened biodiversity is not guaranteed by supplies from lower-income countries at no sustainability (biodiversity, health and food security) cost to the importer country without equitable compensation and support mechanisms. To support this, future research should seek to quantify social and economic pressures associated with more environmentally sustainable foods^54^, including fruits and vegetables, when suitable, compatible spatial data become available.

There are social and economic trade-offs to consumer and policy choices in an increasingly connected global trade network. Our analyses could be interpreted to suggest that imports with high biodiversity pressures on small amounts being traded should be reduced, especially to high-income countries such as the United Kingdom, to ensure sufficient supply to countries struggling to meet their own nutritional needs. In reality, the situation is far more complex. For instance, export prices could be sufficiently high to guarantee funds to purchase food in lower-income supplier countries, and perhaps the employment and economic gains from farming for export are key to preserving sustainable livelihoods. Conversely, sometimes incomes from exports do not achieve their aim of improving living standards and affordability and accessibility of foods for local people^55^. Our work illuminates the complexity of sustainable decision-making and demonstrates the need for shared producer and consumer responsibility for such decisions and their potential impacts^30,56^.

**Extended Data Fig. 1:**
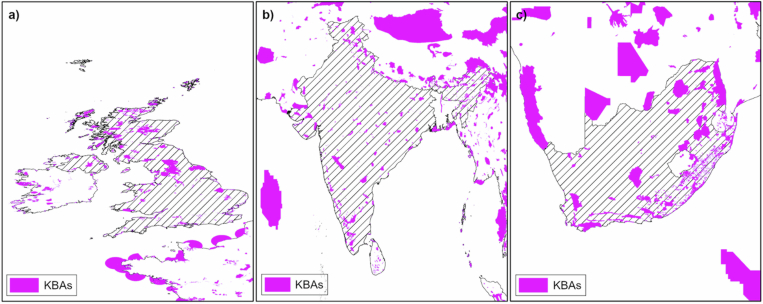
Map of Key Biodiversity Areas in each focal country. **a**) the United Kingdom, **b**) India, and **c**) South Africa. KBAs are designed to capture information on characteristics including: threatened species, geographic restriction, ecological integrity, biological processes, and irreplaceability. These data were supplied by the KBA Partnership^93,94^.

For crops being exported from countries where biodiversity pressures are relatively low, one response could be to increase consumption of these crops over others. However, as the differences between biodiversity-pressure measures show, there is a risk of greater consumption increasing biodiversity pressure, especially in regions where species are already particularly threatened (Extended Data Fig. 1). Moreover, our research demonstrates the importance of trade in influencing the sustainability of different fruits and vegetables with respect to biodiversity. For instance, India’s supply of tomatoes has a lower associated pressure when produced domestically than when supplied by the country’s major trade partner Bangladesh, and therefore if imports of tomatoes from Bangladesh increased in future, this could have implications for increased biodiversity pressure internationally. Similarly, the United Kingdom has increased its production of apples since 2003, a crop that had a lower biodiversity pressure when imported from certain partners (for example, Chile, France and New Zealand) between 1997 and 2003. These examples, and our broader findings, emphasize the importance of monitoring biodiversity pressures as trade partnerships change over time. This would also have the benefit of highlighting crops that prove beneficial to biodiversity, especially if their location used to have a higher-intensity, or more impactful, land use^57^. For example, converting land from an arable farm to an apple orchard could result in lower impact increases to domestic apple production^58^ than we could estimate under our necessary assumption about the negative impacts of cropland on biodiversity. Contrastingly, increasing production could lead to agricultural expansion into new land areas, potentially impacting more biodiversity or displacing other crops with an associated biodiversity pressure change that cannot presently be seen using a biodiversity pressure value estimated at a fixed point in time (for example, here, circa 2000 (1997–2003)). Although monitoring through time could now be possible using our new metrics of biodiversity pressure, it would depend on more up-to-date spatial distribution data for specific crops, and future projections. Our work demonstrates the urgent and important need for such data to be made available.

In addition to the potential risk of ‘hidden’ impacts on biodiversity that some countries might be having internationally via dependency on imports to supply fruits and vegetables, there is also a risk to overall food security. If fruit and vegetable consumption pressures biodiversity, this can threaten the sustainability of ecosystem functioning (by impacting ecosystem-service providers such as pollinator and pest-control species) and, in turn, future agricultural yields^59^. However, populations need to increase their consumption of fruits and vegetables to improve health outcomes^25^. Impacts must therefore be compared with those of other food products to optimize diets for planetary health. It will be important, as higher-resolution data become available, to build on our findings and quantify the relative pressures associated with different foods in different countries to different production systems, land allocations, land-use practices, changing consumption and trade patterns, and local diet types. In addition, future research should attempt to integrate biodiversity pressure with other measures of food system sustainability (for example, greenhouse gas emissions, freshwater consumption, and fertilizer and pesticide use).

Irrespective of where the risk to biodiversity is greatest for a given crop, this risk will be affected by climate change, which is influencing land suitability for fruit and vegetable production and species distributions globally^2,60^. For instance, the United Kingdom’s fruit and vegetable import partners include several of the most climate-change-vulnerable countries according to the ND-GAIN Country Index^61,62^. For example, Kenya is a low-income country ranked 149 (out of 182 countries scored), with a score of 38.7, supplying the United Kingdom (a high-income country, ranked 11th, with a score of 69.4) with foods including pineapples, green beans and peas, avocados, and strawberries. The ND-GAIN Country Index suggests that these supplies might be at risk in future, unless mitigation and adaptation measures are implemented. Quantifying this risk will be an urgent future research aim when sufficient spatialized, crop-specific data become available.

With future climate risks, and our study’s findings, in mind, we posit that we need to broaden the mix of food types and origins consumed to minimize impacts. This will be the case for all foods and could improve the sustainability of future health, food and biodiversity (for example, refs. ^63,64^). One way this could be achieved is by including underutilized crops and indigenous fruit trees to meet the needs of growing fruit and vegetable demands^65–67^. Because our measure of biodiversity is not variety-specific, it is worth noting that the biodiversity pressures associated with these underutilized crops and indigenous fruit trees might not automatically be lower. The realized biodiversity pressure for these will depend on whether native vertebrate fauna have stronger positive associations with these varieties of crops and trees than those presently harvested, which is beyond the scope of this study.

This study highlights trade-offs between multiple Sustainable Development Goals (SDGs)^15^: SDG15, Life on Land; SDG 3, Good Health and Wellbeing; and SDG 12, Responsible Consumption and Production. The previous example of British apple production versus lower biodiversity pressure imports from Chile and New Zealand illustrates trade-offs with other goals too, as imported apples will, potentially, have a larger greenhouse gas and pollution cost^68^ (SDG 13, Climate Action), depending on the time of year of consumption. Another example can be seen in Turkey’s exports of grapes to the United Kingdom, and tomatoes to South Africa and India. These have a lower biodiversity pressure than domestic production, but Turkey is one of the top ten countries in terms of groundwater depletion (SDG 6, Clean Water and Sanitation) associated with global food trade (Table 1 in Dalin et al.^69^). Indeed, India is the highest-ranking country for groundwater depletion^69^. Thus, although from a biodiversity perspective a crop and/or its origin may appear sustainable, without considering other environmental pressures in tandem, it can be difficult to see the true measure of a food’s sustainability.

Our work is subject to a data-driven limitation. We must assume that the spatial coincidence of cropland and biodiversity places a pressure on biodiversity. This is well supported^7^, although it is likely that the intensity of this pressure will vary according to crop, inputs (for example, pesticides and fertilizers) and growing system^70^, with fruits grown on trees, such as apples and plums, likely to provide more diverse and suitable habitats for vertebrates than low-growing fruits such as pineapples, for example, and some fruits and vegetables being grown with more pesticides and herbicides than others. Biodiversity varies according to landscape configuration, composition and heterogeneity, and is affected by farm sizes and farming intensity^28,70–74^. African farms are typically smaller and lower input than in other world regions and might therefore harbour more biodiversity^74,75^, but global-scale, crop-specific farm-size data are not yet available to investigate this influence on the relationships we identify in South Africa and its African trade partners^76^. We also cannot separate crops being grown under glass from those in open air. Our data also represent the year 2000, but no other more recent data exist to map the distributions of more than a handful of health-relevant fruit and vegetable crops. Our results provide a useful indicator of the potential pressures placed by fruits and vegetables on biodiversity and where risks are greatest but due to data resolution should not be used to pinpoint pressures on specific species or fields driven by specific crops. Future work is needed to ‘ground-truth’ our findings, which provide insights into countries producing fruits and vegetables with especially high biodiversity pressures that could be prioritized for field studies.

In sum, there are no clear ‘best’ fruits or vegetables to choose for a ‘win–win’ for biodiversity and human health. Humans, overall, need more fruits and vegetables in their diet than they have at present. Our work emphasizes the importance of accounting for the role of trade (supply and demand sides) in influencing the sustainability of essential food supplies, especially for countries ‘outsourcing’ biodiversity impacts. We highlight the need for per-unit measures of biodiversity pressure to be incorporated into decisions on current and future land use and food-system sustainability, alongside more common measures of greenhouse gas emissions and water use. Our spatialized metrics and approach can be used to evaluate the biodiversity pressures associated with other crops and can be incorporated into integrated assessment models. For fruits and vegetables specifically, we must work to find sustainable production methods and sources of those consumed most, according to country-specific palates, nutritional needs, threatened biodiversity, trade partnerships, climate vulnerabilities and land suitability for domestic production.

## Methods

### Global production and consumption of fruit and vegetable crops

We focus this work on fruit and vegetable consumption in the United Kingdom, India and South Africa. These countries are not meeting their fruit and vegetable consumption guidelines, as is the case for many countries, globally. The World Health Organization recommends 400 g of fruits and vegetables per day^25^. According to means taken across all age groups, education levels and residence types, and both sexes, from the Global Dietary Database records^77,78^ for fruits and non-starchy vegetables for 1990–2018, only 91 g of fruits and 118 g of vegetables are consumed on average per day in the United Kingdom, 37 g of fruits and 166 g of vegetables on average per day in India, and 60 g of fruits and 134 g of vegetables on average per day in South Africa, suggesting that the populations in these countries are consuming only around half of the recommended quantities of fruits and vegetables.

Our analysis is global in scope: we measure the pressure being placed on biodiversity by fruits and vegetables consumed in the United Kingdom, India and South Africa, and compare pressures associated with different international trade partnerships, and with domestic production. The crop data we use^79,80^ are freely available, so our findings can, in future, be replicated in other countries and extended for other purposes in the focal countries.

We use Monfreda et al.’s^79,80^ crop harvested area and yield data from circa 2000 because these include 50 individual fruit and vegetable crops also recorded in the trade data we used and are spatialized for these crops on a global scale using subnational-scale survey and gridded remote-sensing (satellite) data. These data also have a similar timeframe to available biodiversity data (described below). The original Monfreda et al.^79,80^ dataset includes 61 fruit and vegetable crops, but this number includes six groups of mixed fruits and vegetables (unspecified ‘berries’, ‘citrus fruit’, ‘fresh fruit’, ‘stone fruit’, ‘tropical fruit’ and ‘vegetables’). We further exclude five crops (green onions, ‘melon etc.’ (other melons, including canteloupes), peppers, cashew apples and string beans) which are not consumed by the focal countries and/or do not have associated records for the trade data described below.

**Extended Data Fig. 2:**
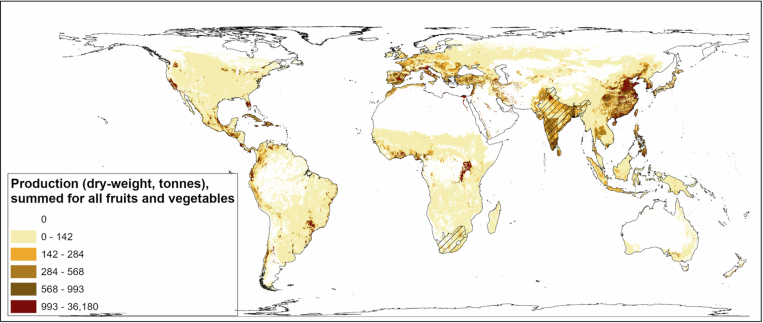
Map of global production. (from Monfreda et al.^79^), with hashed lines highlighting the focal countries (UK, India, and South Africa).

Given the data we used, our results cannot capture land-use changes that have taken place in the last 15–20 years, but do indicate which countries and regions are under greater relative biodiversity pressure than others, and which crops and trade relationships are driving this. Other freely available, spatialized and more recent data are not available for a variety of fruits and vegetables. For instance, SPAM^81^ contains more recent global data (2010), but only covers four specific fruits and vegetables and three groups—tropical fruit, temperate fruit and vegetables. Monfreda et al.’s^79,80^ data are available for 31 fruits and 24 vegetables, and include grouped categories we exclude from our analysis due to their non-specificity. The resolution of the Monfreda et al.^79,80^ data selected is 5 arcmin (∼9 km at the equator), and the data cover the period 1997–2003. We used the R^82^ package raster^83^ to ensure that the extent and projection of the crop and biodiversity data were consistent, conducting bilinear resampling, appropriate for continuous data, of the crop data to the 5-arcmin (~9 km at the equator) resolution species data. Production data are mapped, for reference, in Extended Data Fig. 2.

Dry-weight production values were used, calculated for each fruit and vegetable analysed using water-content averages from food composition data from the USDA National Nutrient Database for Standard ref. ^84^ (averages used as given in Supplementary Table 2). Dry-weight production values were calculated by multiplying the raster for each fruit and vegetable by 1 − water content (g)/100.

In our work, where consumption is not split into domestic and imported components (for example, Fig. 2), consumption is defined as production plus imports minus exports (all in tonnes), also commonly termed ‘supply’. We processed trade data from the Food and Agriculture Organization (FAO) to measure consumption. To determine the overall movement of food and agricultural products, processed foods were converted into primary crop equivalents, as in Dalin et al.^69^, based on FAO product categorization. FAO production and trade values were adjusted using a balancing algorithm, to link final demand to origin of primary product, created by Kastner et al.^69,85^. This algorithm converts secondary products, derived from primary products, into their primary equivalents using data on primary product production and bilateral trade, secondary products derived from these primary products, calorie contents and extraction rates^69,85,86^.

We used average traded metric tonnes of primary equivalent crops for 1997–2003 (to match the crop data period represented by Monfreda et al.^79,80^). We also assessed changes in trade (in tonnes) since 2003 (up to 2017, which was the most recent year for which country trade data were reliable and complete) to produce Fig. 5.

### Vertebrate richness mapping

**Extended Data Fig. 3:**
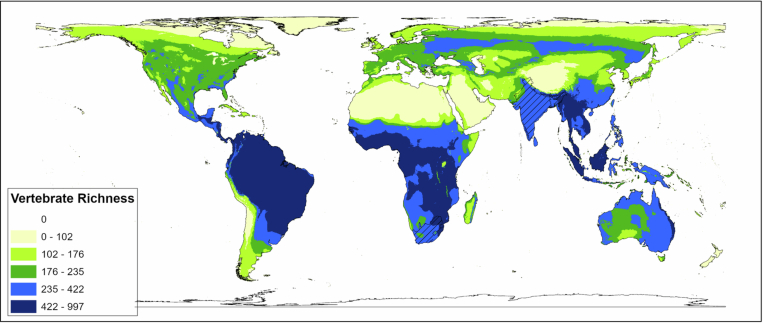
Map of global ‘raw’ vertebrate species richness. (Etard et al.^87^), with hashed lines highlighting the three focal countries (UK, India, and South Africa).

We represent biodiversity using species richness—the most readily available biodiversity metric globally for the largest number of vertebrate species. We focus on vertebrate (mammal, bird, amphibian and reptile) species richness because these are the best-sampled taxonomic groups globally and have been the focus of studies that have demonstrated significant negative relationships between human land uses (including cropland) and species richness, which is a key assumption of our work. We use 10-km resolution, global-scale, spatialized species-richness estimates from Etard et al.^87^ comprising species records contributed in circa 2012. These maps were produced by summing species-range distribution maps from BirdLife International^88^ and the IUCN^89^. These organisations also provide the information to facilitate the exclusion of areas outside of known elevational limits for species, carried out for these data. Using range maps to estimate species richness is appropriate for representing the broad distribution of species across the globe and within a given country, despite known limitations^90^ (for example, assuming that a species is present across its entire range and excluding species which have become extinct since ranges were last recorded). Vertebrate richness data are shown, for reference, in Extended Data Fig. 3.

Our work makes the necessary assumption that the production of fruits and vegetables places a pressure on vertebrate richness because our new biodiversity pressure indicators (described next) quantify the overlap between cultivated areas and species ranges. This assumption has also been made^48^ and tested^7^ in previously published studies.

### Biodiversity pressure of fruits and vegetables by country and crop

We created a new measure, ASR (species·ha), to encapsulate the actual area occupied by specific crops in a country and the number of vertebrate species (hereafter ‘species’) in this area potentially being put under pressure by this. This is a per-grid-cell and per-crop measure, rather than per tonne, and therefore reflects the overall biodiversity pressured area associated with a given crop.

The ASR is defined as follows:

1 $${\mathrm{ASR}}={\mathrm{HA}}_{c,l}\times {\mathrm{SR}}_{l}$$

where HA_*c*,*l*_ is the harvested area of crop *c* at location (that is, a 5-arcmin grid cell) *l* (average number of hectares harvested per land-area of a grid cell, from Monfreda et al.^79,80^; cell area = 0.083333°^2^, or ~1,000 ha at the equator) and SR_*l*_ is the vertebrate species richness at location *l* (that is, the number of species, computed as described in ‘Vertebrate richness mapping’).

We mapped the ASR in each grid cell using the raster package^83^ in R^82^, enabling us to identify pressure hotspots associated with fruits and vegetables being grown in our focal countries and their trade partner countries. We also quantified the weighted-mean ASR (weighted by dry-weight production in tonnes) for each fruit and vegetable for each country in the world (except Greenland and Antarctica) using the exactextract package^91^ in R^82^.

### Biodiversity pressure per tonne of crop under current environmental and crop-management conditions

We developed a biodiversity pressure intensity metric (BP) that builds on the ASR to capture the number of vertebrate species (or species richness) potentially impacted by cropland per hectare per tonne of a given crop. This enables us to compare crops, considering their pressures according to global harvests in general, rather than specific pressures placed by a given country’s consumption. We designed this metric to be fully scalable but also relativized to the amount of crop being produced in the area for which it is quantified. This is important because we are assessing the trade-off between wild animal health and human health; a greater amount of fruit and vegetable produced implies more potential benefit to human health by supporting a supply of healthy foods, whereas a large extent of cropland in a biodiverse area will put more pressure on wild animal health. Accordingly, a fruit or vegetable that puts a high pressure on biodiversity (high ASR) but provides nutrition for many people (high production value) would have a lower BP value than one that has the same ASR but is associated with low levels of food production.

The production-side biodiversity pressure metric, BP, at the grid-cell level is defined as follows:

2 $${\mathrm{BP}}_{c,l}=\frac{\left({\mathrm{HA}}_{c,l}\times {\mathrm{SR}}_{l}\right)}{{P}_{c,l}}$$

where HA_*c*,*l*_ is the harvested area of crop *c* at location *l* (that is, the average number of hectares harvested per land-area of a grid cell, from Monfreda et al.^79,80^), SR_*l*_ is the vertebrate species richness at location *l* (that is, the number of species, computed as described in ‘Vertebrate richness mapping’), and *P*_*c*,*l*_ is the production of crop *c* at location *l* in tonnes. Production was calculated using the product of yield (t ha^−1^) and harvested area (ha) at location *l*, both of which are datasets available at the same resolution and for the same crops with global, spatialized coverage via Monfreda et al.^79,80^.

As well as mapping BP per grid cell using the R package raster^82,83^, we quantified the average pressure for each fruit and vegetable for each country in the world (except Greenland and Antarctica) using the R package exactextract^82,91^. The average of production-side BP (weighted by dry-weight production) across all grid cells in each country was computed as follows:

3 $${\mathrm{BP}}_{c,i}=\frac{\sum _{l\,{\mathrm{in}}\,i}{P}_{c,l}\times {\mathrm{BP}}_{c,l}}{\sum _{l\,{\mathrm{in}}\,i}{P}_{c,l}}$$

where BP_*c*,*i*_ is the biodiversity pressure of crop *c* in country *i* and *P*_*c*,*l*_ is the dry-weight production of crop *c* at location *l* in tonnes, so this equation sums over cells *l* in country *i* to get the country-level, weighted-average BP, presented in figures summarizing imports (Figs. 2b and 3b). The weighting means that, when combining with international trade data (equation (5)), we consider that the origins of exported crops are distributed in the same way across grid cells as production in a country (that is, exports are more likely to come from locations where production is the largest).

The biodiversity pressure associated with national production (BP_prod_) sums biodiversity pressure over a country, as follows:

4 $${\mathrm{BP}}_{\mathrm{prod}\,{c,i}}={\mathrm{BP}}_{c,i}\times {P}_{c,i}$$

BP_prod_ is presented in Figs. 2b and 3b for each focal country’s domestic component of BP.

### Biodiversity pressures associated with national consumption of fruits and vegetables

Finally, we developed a measure of biodiversity pressure associated with national consumption, BP_cons_, to represent the pressure on biodiversity embedded in the consumption of a country (here, the United Kingdom, India and South Africa). BP_cons_ accounts for pressures associated with a country’s consumption, made up of domestically produced and imported food.

BP_cons_ is calculated as follows:

5 $${\mathrm{BP}}_{\mathrm{cons}\,{{\mathrm{fc}},c}}={C}_{\mathrm{fc},c}^{\;\mathrm{dom}}\times {\mathrm{BP}}_{\mathrm{fc},c}+\sum _{i}{T}_{i,{\mathrm{fc}},c}\times {{BP}}_{c,i}$$

where BP_cons fc,*c*_ is the biodiversity pressure associated with the national consumption of the focal country (fc) and crop *c*, ${C}_{\mathrm{fc},c}^{\mathrm{dom}}$ is the local, domestic supply (in tonnes) of crop *c* to the focal country (production − exports), *T*_*i*,fc,*c*_ is the import of crop *c* (in tonnes) from country *i* to the focal country (where *i* is an import partner), and BP_*c*,*i*_ is the average biodiversity pressure computed at the country level as described in equation (3).

By providing multiple measures of biodiversity pressure, and data on consumption and trade, we can start to see whether a high pressure is due to high biodiversity in a country overall, large amounts of a crop being produced/consumed, or because the risk of a crop is particularly high due to where it is grown within a country, relative to species ranges within/across countries.

### Reporting summary

Further information on research design is available in the Nature Portfolio Reporting Summary linked to this article.

## Supplementary information

Supplementary Information Supplementary Tables 1.1-1.3 descriptions, Supplementary Table 1.4, Supplementary Table 2 description and Description of R scripts and data.

Reporting Summary

Supplementary Table 1.1 Supplementary Table 1.1: Excel file with tabs for: i) total vertebrate species richness per country; ii) maximum vertebrate species richness per country; iii) mean affected species range (ASR) per country (all fruits and vegetables); iv) total production per country (dry-weight, all fruits and vegetables, tonnes); v) mean biodiversity pressure (BP) per country (species * ha/tonnes, all fruits and vegetables); vi) ISO3 codes matched up to countries as named in the trade and R ‘worldmap’96 datasets; vii) global ranking of fruits and vegetables according to global BP; viii) global rankings of fruits and vegetables according to global production; ix) global rankings of fruits and vegetables according to ASR.

Supplementary Table 1.2 Supplementary Table 1.2: Excel file with: i) mean biodiversity pressure (BP) per crop per country.

Supplementary Table 1.3 Supplementary Table 1.3: Excel file with: i) biodiversity pressure associated with national consumption (BPcons) per crop for each focal country.

Supplementary Table 2 Supplementary Table 2: Excel file with the water content averages used to calculate dry-weight production.

## Data Availability

The data used as inputs for the analyses in this manuscript are openly available for download and cited in the manuscript. Data produced in this study are available as Supplementary Data Files on GitHub via: https://github.com/abbiesachapman/SHEFS_FV.

## Code availability

The R scripts created and used for the analyses in this study are provided as Supplementary Files available on GitHub via: https://github.com/abbiesachapman/SHEFS_FV. The approach for producing the trade data used from freely available FAO data is described in the Methods and the references cited therein. Code for this is available upon request.
