# Supplementary material for: Biodiversity pressure from fruit and vegetable consumption in the United Kingdom, India and South Africa varies by product and growing location
Source: Nat Food. 2025 Aug 25;6(9):892–905. doi: 10.1038/s43016-025-01222-y (PMC12454118; doi:10.1038/s43016-025-01222-y)
Supplement: Supplementary file 1 — Supplementary Tables 1.1-1.3 descriptions, Supplementary Table 1.4, Supplementary Table 2 description and Description of R scripts and data. [file 43016_2025_1222_MOESM1_ESM.pdf]

nature food

Supplementary information

https://doi.org/10.1038/s43016-025-01222-y

Biodiversity pressure from fruit and vegetable consumption in the United Kingdom, India and South Africa varies by product and growing location

In the format provided by the authors and unedited

SUPPLEMENTARY INFORMATION

Supplementary Table 1.1: Excel file with tabs for: i) total vertebrate species richness per country; ii) maximum vertebrate species richness per country; iii) mean affected species range (ASR) per country (all fruits and vegetables); iv) total production per country (dry-weight, all fruits and vegetables, tonnes); v) mean biodiversity pressure (BP) per country (species * ha/tonnes, all fruits and vegetables); vi) ISO3 codes matched up to countries as named in the trade and R 'worldmap'[96] datasets; vii) global ranking of fruits and vegetables according to global BP; viii) global rankings of fruits and vegetables according to global production; ix) global rankings of fruits and vegetables according to ASR.

Supplementary Table 1.2: Excel file with: i) mean biodiversity pressure (BP) per crop per country.

Supplementary Table 1.3: Excel file with: i) biodiversity pressure associated with national consumption (BP_{cons}) per crop for each focal country.

Supplementary Table 1.4: Table of mean and median values for dry-weight consumption (tonnes), per-tonne biodiversity pressure (BP, species.hectares (ha) /tonne), and biodiversity pressure associated with national consumption (BP_{cons}, species.hectares (ha)), to accompany **Fig. 2.** Domestic is abbreviated to 'Dom.', Imported to 'Imp.', Vegetables to 'Veg.' and United Kingdom to 'UK' for display purposes. The higher value of fruits and vegetables in each domestic and imported group is underlined and the overall highest value per country is emboldened. The proportion of each country's consumption of fruits and vegetables from domestic and imported sources is presented in the 'Consumption' section of this table in brackets, next to each type of fruit and vegetable.

Metric	Country	Dom. /Imp.	Fruits / Veg.	Median	Mean
Consumption (tonnes)	UK	Dom.	Fruits (18%)	6	9,450
			Veg. (52%)	13	**48,380**
		Imp.	Fruits (82%)	0.02	1,464
			Veg. (48%)	0.03	1,411
	India	Dom.	Fruits (99%)	1	558,969
			Veg. (99.9%)	1	**1,140,186**
		Imp.	Fruits (0.75%)	0.00000197	173
			Veg. (0.09%)	0.00000599	51
	South Africa	Dom.	Fruits (99%)	1	**62,884**
			Veg. (99%)	6	46,534
		Imp.	Fruits (0.3%)	0.000000961	7
			Veg. (1%)	0.00000632	21
BP (species.ha /tonne)	UK	Dom.	Fruits	143	188

			Veg.	49	130
		Imp.	Fruits	138	**40,092,822,638,848,516,096**
			Veg.	74	160
	India	Dom.	Fruits	174	182
			Veg.	313	417
		Imp.	Fruits	168	**41,259,222,767**
			Veg.	199	345
	South Africa	Dom.	Fruits	179	320
			Veg.	268	444
		Imp.	Fruits	202	**2,786,016,105,886**
			Veg.	183	416
BP_{cons} (species.ha)	UK	Dom.	Fruits	1,721,314	2,741,814
			Veg.	7,751,539	10,656,406
		Imp.	Fruits	7,763,873	**6,511,260,884,339,119,882,440**
			Veg.	2,963,724	9,243,690
	India	Dom.	Fruits	3,474,630	191,524,150
			Veg.	314,384,051	601,524,351
		Imp.	Fruits	25,939	**8,654,077,975,066,261**
			Veg.	20,522	397,706
	South Africa	Dom.	Fruits	6,551,577	20,982,137
			Veg.	16,694,419	27,819,595
		Imp.	Fruits	22,303	**1,823,226,046,808,078**
			Veg.	16,902	168,954

Supplementary Table 2: Excel file with the water content averages used to calculate dry-weight production.

R scripts and data: R scripts and data are available on GitHub as described in the main manuscript.
