# Supplementary material for: Biodiversity pressure from fruit and vegetable consumption in the United Kingdom, India and South Africa varies by product and growing location
Source: Nat Food. 2025 Aug 25;6(9):892–905. doi: 10.1038/s43016-025-01222-y (PMC12454118; doi:10.1038/s43016-025-01222-y)
Supplement: Supplementary file 2 — Reporting Summary [file 43016_2025_1222_MOESM2_ESM.pdf]

nature portfolio

Corresponding author(s): Abbie S.A. Chapman

Last updated by author(s): 2025/07/17

Reporting Summary

Nature Portfolio wishes to improve the reproducibility of the work that we publish. This form provides structure for consistency and transparency in reporting. For further information on Nature Portfolio policies, see our Editorial Policies and the Editorial Policy Checklist.

Statistics

For all statistical analyses, confirm that the following items are present in the figure legend, table legend, main text, or Methods section.

n/a	Confirmed	
☒	☐	The exact sample size (*n*) for each experimental group/condition, given as a discrete number and unit of measurement
☒	☐	A statement on whether measurements were taken from distinct samples or whether the same sample was measured repeatedly
☒	☐	The statistical test(s) used AND whether they are one- or two-sided<br>*Only common tests should be described solely by name; describe more complex techniques in the Methods section.*
☒	☐	A description of all covariates tested
☒	☐	A description of any assumptions or corrections, such as tests of normality and adjustment for multiple comparisons
☐	☒	A full description of the statistical parameters including central tendency (e.g. means) or other basic estimates (e.g. regression coefficient) AND variation (e.g. standard deviation) or associated estimates of uncertainty (e.g. confidence intervals)
☒	☐	For null hypothesis testing, the test statistic (e.g. *F*, *t*, *r*) with confidence intervals, effect sizes, degrees of freedom and *P* value noted<br>*Give P values as exact values whenever suitable.*
☒	☐	For Bayesian analysis, information on the choice of priors and Markov chain Monte Carlo settings
☒	☐	For hierarchical and complex designs, identification of the appropriate level for tests and full reporting of outcomes
☒	☐	Estimates of effect sizes (e.g. Cohen's *d*, Pearson's *r*), indicating how they were calculated

Our web collection on statistics for biologists contains articles on many of the points above.

Software and code

Policy information about availability of computer code

Data collection	Data used for the analysis include: trade data for the years 1997-2017 from the Food and Agricultural Organisation (accessible at https://fao.org/faostat/en/#data); crop harvested area and yield data representing c.2000 available from http://www.earthstat.org/harvested-area-yield-175-crops/ (Monfreda et al., 2008, doi: 10.1029/2007GB002947, downloaded 12/07/19); water-content data for fruits and vegetables available from the USDA National Nutrient Database for Standard Reference (https://doi.org/10.15482/USDA.ADC/1324304, release 28, accessed 21/10/22); and vertebrate species richness maps produced as described in Etard et al. (2020; doi: 10.1111/geb.13184) from freely available sources (Roll et al., 2017, https://datadryad.org/stash/dataset/doi:10.5061/dryad.83s7k (downloaded 13 April 2020); IUCN Red List (IUCN, 2020, downloaded 13 April 2020); and BirdLife International (http://datazone.birdlife.org/species/requestdis, downloaded 17 April 2020)).
Data analysis	All analyses are documented in full in the manuscript (and references therein) and citations are provided for software and packages used (R and ArcGIS). R code is available on GitHub. All data produced in this study are available on figshare. The approach for producing the trade data from FAO data is described in the manuscript and the references cited therein. Code for this is available upon request.

For manuscripts utilizing custom algorithms or software that are central to the research but not yet described in published literature, software must be made available to editors and reviewers. We strongly encourage code deposition in a community repository (e.g. GitHub). See the Nature Portfolio guidelines for submitting code & software for further information.

Data

Policy information about availability of data

All manuscripts must include a data availability statement. This statement should provide the following information, where applicable:

- Accession codes, unique identifiers, or web links for publicly available datasets
- A description of any restrictions on data availability
- For clinical datasets or third party data, please ensure that the statement adheres to our policy

The data used as inputs for the analyses in this manuscript were openly available for download, as described and cited in the manuscript. Data produced in this study are available as Supplementary Data Files on figshare. The R scripts created and used for the analyses in this study are available on GitHub. The approach for producing the trade data used from freely available FAO data is described in Online Methods and the references cited therein. Code for this is available upon request.

Research involving human participants, their data, or biological material

Policy information about studies with human participants or human data. See also policy information about sex, gender (identity/presentation), and sexual orientation and race, ethnicity and racism.

Reporting on sex and gender	This research does not involve human research participants.
Reporting on race, ethnicity, or other socially relevant groupings	This research does not involve human research participants.
Population characteristics	This research does not involve human research participants.
Recruitment	This research does not involve human research participants.
Ethics oversight	This research does not involve human research participants.

Note that full information on the approval of the study protocol must also be provided in the manuscript.

Field-specific reporting

Please select the one below that is the best fit for your research. If you are not sure, read the appropriate sections before making your selection.

☐ Life sciences ☐ Behavioural & social sciences ☒ Ecological, evolutionary & environmental sciences

For a reference copy of the document with all sections, see nature.com/documents/nr-reporting-summary-flat.pdf

Ecological, evolutionary & environmental sciences study design

All studies must disclose on these points even when the disclosure is negative.

Study description	A spatial analysis quantifying the biodiversity pressures associated with fruit and vegetable crops consumed in the United Kingdom, South Africa, and India.
Research sample	This study is a global analysis as we measure pressures associated with international trade partnerships and the data we used are at the 5 arcmin (~10 km at the equator) resolution.
Sampling strategy	The study design is a spatial analysis using open, map data and therefore did not require sampling.
Data collection	We used publicly available data sources (FAOSTAT; Monfreda et al., 2008; USDA; Roll et al, 2017; IUCN; and BirdLife International) for our analysis. Data used for the analysis include: trade data for the years 1997-2017 from the Food and Agricultural Organisation (accessible at https://fao.org/faostat/en/#data); crop harvested area and yield data representing c.2000 available from http://www.earthstat.org/harvested-area-yield-175-crops/ (Monfreda et al., 2008, doi: 10.1029/2007GB002947, downloaded 12/07/19); water-content data for fruits and vegetables available from the USDA National Nutrient Database for Standard Reference (https://doi.org/10.15482/USDA.ADC/1324304, release 28, accessed 21/10/22); and vertebrate species richness maps produced as described in Etard et al. (2020; doi: 10.1111/geb.13184) from freely available sources (Roll et al., 2017, https://datadryad.org/stash/dataset/doi:10.5061/dryad.83s7k (downloaded 13 April 2020); IUCN Red List (IUCN, 2020, downloaded 13 April 2020); and BirdLife International (http://datazone.birdlife.org/species/requestdis, downloaded 17 April 2020)).
Timing and spatial scale	The trade data used span 1997-2017. The crop data represent c.2000, but span 1997-2003 (see Monfreda et al., 2008, for a complete data description). The vertebrate species data are comprised of records from a range of years, released in the years as cited to represent the best available understanding of species distributions at the time (2017 for reptiles, 2020 for mammals and amphibians, and 2020 for birds). For our analyses, we work at a global scale with a cell resolution of 5 arcmin (~10km at the equator).

Data exclusions	Data on specific crops were excluded if the crops were not being grown in, or consumed in, the UK, South Africa, and/or the UK.
Reproducibility	The study design was not experimental.
Randomization	The study design was a spatial analysis so did not require randomization.
Blinding	The study design was a spatial analysis so did not require blinding.

Did the study involve field work? ☐ Yes ☒ No

Reporting for specific materials, systems and methods

We require information from authors about some types of materials, experimental systems and methods used in many studies. Here, indicate whether each material, system or method listed is relevant to your study. If you are not sure if a list item applies to your research, read the appropriate section before selecting a response.

Materials & experimental systems

n/a	Involved in the study
☒	☐ Antibodies
☒	☐ Eukaryotic cell lines
☒	☐ Palaeontology and archaeology
☒	☐ Animals and other organisms
☒	☐ Clinical data
☒	☐ Dual use research of concern
☒	☐ Plants

Methods

n/a	Involved in the study
☒	☐ ChIP-seq
☒	☐ Flow cytometry
☒	☐ MRI-based neuroimaging

Plants

Seed stocks	NA
Novel plant genotypes	NA
Authentication	NA
